# SEB genotyping: SmartAmp-Eprimer binary code genotyping for complex, highly variable targets applied to HBV

**DOI:** 10.1186/s12879-022-07458-4

**Published:** 2022-06-03

**Authors:** Diane Delobel, Yutaka Furutani, Sumiko Nagoshi, Akihito Tsubota, Akio Miyasaka, Koichi Watashi, Takaji Wakita, Tomokazu Matsuura, Kengo Usui

**Affiliations:** 1 Genetic Diagnosis Technology Unit, RIKEN Center for Integrative Medical Sciences, Yokohama, Kanagawa Japan; 2 Liver Cancer Prevention Research Unit, RIKEN Cluster for Pioneering Research, Wako, Saitama 351-0198 Japan; 3 Department of Gastroenterology and Hepatology Saitama Medical Center, Saitama Medical University, Saitama, 350-8550 Japan; 4 Core Research Facilities, Research Center for Medical Science, The Jikei University School of Medicine Minato-Ku, Tokyo, 105-8461 Japan; 5 Division of Hepatology, Department of Internal Medicine, Iwate Medical University, School of Medicine, Shiwa-gun, Iwate, 028-3695 Japan; 6 Department of Virology II, National Institute of Infectious Diseases, Tokyo, Japan; 7 Department of Applied Biological Science, Tokyo University of Science, Noda, Japan; 8 Institute for Frontier Life and Medical Sciences, Kyoto University, Kyoto, Japan; 9 MIRAI, JST, Saitama, Japan; 10 Research Center for Drug and Vaccine Development, National Institute of Infectious Diseases, Tokyo, Japan; 11 National Institute of Infectious Diseases, Tokyo, Japan; 12 Department of Laboratory Medicine, The Jikei University School of Medicine, Minato-ku, Tokyo 105-8461 Japan; 13 Nucleic Acid Diagnostic System Development Unit, RIKEN Center for Integrative Medical Sciences, Yokohama, Kanagawa Japan

**Keywords:** Isothermal amplification, Genotyping, Hepatitis B virus, High mutation rate, Infectious diseases

## Abstract

**Background:**

SmartAmp-Eprimer Binary code (SEB) Genotyping is a novel isothermal amplification method for rapid genotyping of any variable target of interest.

**Methods:**

After in silico alignment of a large number of sequences and computational analysis to determine the smallest number of regions to be targeted by SEB Genotyping, SmartAmp primer sets were designed to obtain a binary code of On/Off fluorescence signals, each code corresponding to a unique genotype.

**Results:**

Applied to HBV, we selected 4 targets for which fluorescence amplification signals produce a specific binary code unique to each of the 8 main genotypes (A–H) found in patients worldwide.

**Conclusions:**

We present here the proof of concept of a new genotyping method specifically designed for complex and highly variable targets. Applied here to HBV, SEB Genotyping can be adapted to any other pathogen or disease carrying multiple known mutations. Using simple preparation steps, SEB Genotyping provides accurate results quickly and will enable physicians to choose the best adapted treatment for each of their patients.

**Supplementary Information:**

The online version contains supplementary material available at 10.1186/s12879-022-07458-4.

## Background

Genetic variation between individuals within a population (viral, bacterial or even in humans) can be silent, lead to different phenotypes, diseases or death if it occurs in essential genes. Genetic variants can be found either at single nucleotide location or in more complex repartition schemes over a gene. Examples of viruses with complex genetic variants are the human papillomavirus (HPV) where out of over 170 closely related types, HPV-type 16 and 18 lead to over 60% of HPV-related cancers [1, 2] or the hepatitis B virus (HBV). HBV is a leading cause of liver cancer, resulting in over 880,000 deaths per year [3]. Divided into 10 genotypes (A–J) of which 5 cause over 96% of infections worldwide [respectively genotype C (26%), D (22%), E (18%), A (17%) and B (14%)] [4], it has been extensively studied that HBV genotypes have an influence on evolution or prognosis of the liver diseases, risks of complications, and responses to treatment [5–7].

While sequencing is considered the gold standard for the detection of all genetic variations, its cost and preparation time are often cited as major limitation for its usage [8, 9]. Isothermal amplification methods have been developed that are as sensitive as PCR while producing results much faster at low cost, making them easy and attractive tools for small point of care settings or developing regions, as recently mentioned in multiple reviews [10–13]. SmartAmp is an isothermal nucleic acid amplification method [14] that can be combined with sequence-specific probes such as Exciton-Controlled Hybridization-sensitive fluorescent Oligonucleotides (ECHOs) for genotyping [15–17]. Called “Exciton probe—Eprobe” [18, 19] or “Exciton primer—Eprimer”, ECHOs are oligonucleotides that only emit fluorescence upon binding to their sequence specific targets. So far, SmartAmp has been used for SNP genotyping (wild-type vs mutant) either with one [20] or duplex Eprimer labelling [21–23].

We hypothesized that combining Eprimer On/Off fluorescence signal detection into a specific, digitized or binary code would enable us to distinguish and identify complex genetic variants. To realize this concept, we applied our analysis results to HBV genotyping. Historically, most HBV genotyping methods based on nucleic acid amplification and detection have focused on the highly conserved pre-S/-S gene region to distinguish between different HBV genotypes and sub-genotypes [5]. Utilizing this region, we developed our new SmartAmp binary code genotyping primer sets for HBV. Here, we describe a new usage of SmartAmp-Eprimer, combining in silico alignment of a large number of sequences and computational analysis to determine the smallest number of regions targeted for amplification, leading to a binary code of On/Off fluorescence signals, each code corresponding to a unique genotype. We named this new technique “SmartAmp-Eprimer Binary code Genotyping (SEB Genotyping)”.

## Methods

### HBV sequence alignments

**Fig. 1:**
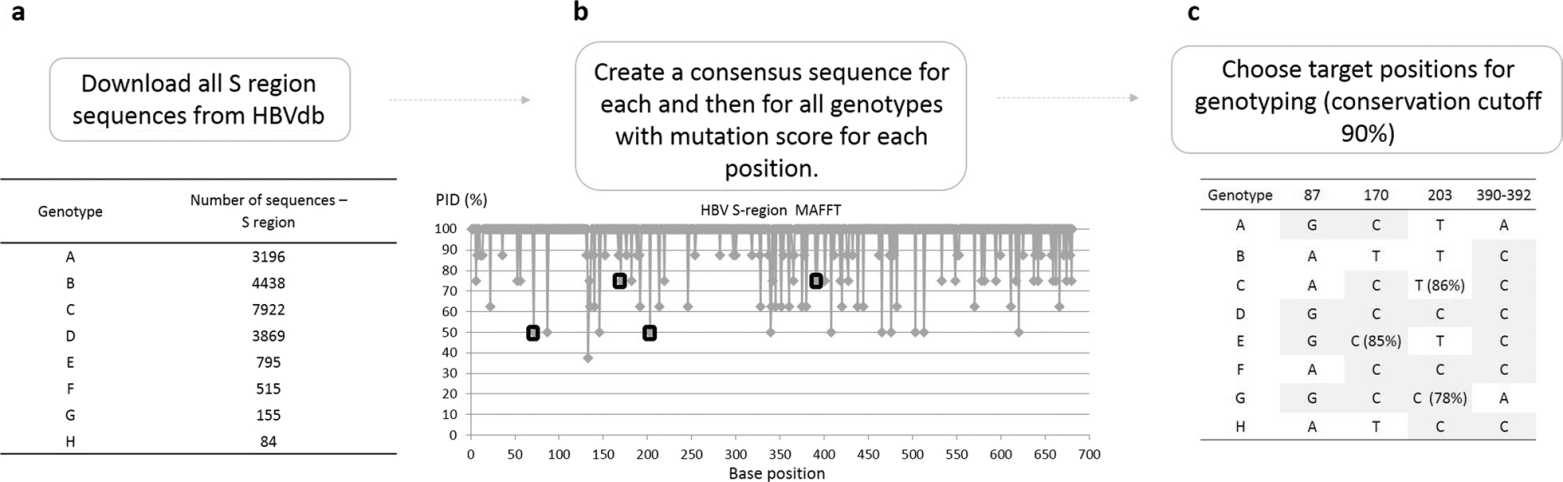
Flowchart to determine mutation positions used for genotyping. **a** FASTA sequences for the S region of HBV were downloaded from HBVdb and aligned into a sequence. **b** For each position a mutation score was calculated and (**c**) targets were selected that are > 90% conserved within one genotype but different between different genotypes

Alignment of HBV sequences followed a protocol similar to the one described in [24] but revised by us for large data analysis. All the FASTA sequences for the S region of HBV sorted by genotype A to H available on the HBV database (HBVdb) [25] website were downloaded (https://hbvdb.lyon.inserm.fr/HBVdb/HBVdbDataset?seqtype=0 accessed May 8th 2018). For each of the 8 genotypes, all the sequences available (shown in Fig. 1a) were aligned to create a consensus sequence using MAFFT on Jalview desktop software (Jalview 2.10.4) and their PIDs (percentage of identity between the multiple sequences) were exported [26]. These 8 consensus sequences were then aligned to create a pan-genotype consensus sequence and PID, using the aforementioned method. Results are shown in Additional file 1. Using the alignment of 8 consensus sequences instead of the global alignment of over 20,000 HBV sequences permitted us to reduce the bias against rare genotypes (Additional file 2).

### Selection of the target positions for genotyping

For each position in the pan-genotype sequence (Fig. 1b), the PID values were analyzed to identify nucleotides that were over 90% conserved within one genotype but differed between genotypes: for example, at the position 87 genotype A is 96.43% conserved as G but genotype C is 98.08% conserved as A. Then the minimum combination of specific nucleotide positions that permitted discriminating between genotypes was manually selected. Because we were targeting a relatively small (681 bp) and highly conserved region, this was done manually using Excel. In our dataset, all 8 genotypes could be identified using 4 targeted nucleotide positions (Fig. 1c, PID per genotype > 90% or otherwise noted).

### SmartAmp primer sets design

SmartAmp primer sets were designed as previously described [14, 20] to amplify the regions surrounding the four selected target positions. Following the recommendations described by Kimura et al. [17, 27], Eprimers were carefully designed so that (i) the ECHO-labeled thymidine (marked Z in Table 1) was not placed in the last two positions at the 5′ or 3′ end of the oligonucleotide, (ii) it was not surrounded by a mismatch within 2 bases either in the 5′ or 3′ direction, and (iii) the four targeted bases for genotyping (i.e., positions 87, 170, 203 and 390–392 of our pan-genotype consensus sequence) were placed as the penultimate 3′-end nucleotide of each Eprimer for On/Off effect. Eprimers were purchased from DNAFORM (Yokohama, Japan), and DNA oligonucleotides were purchased from Eurofins Genomics (Tokyo, Japan) or Sigma-Aldrich (Tokyo, Japan). Sequences for standard oligonucleotides and Eprimers (noted oBP) are listed in Table 1.

**Table 1:** SmartAmp primer sequences

Target position	Primer name	Sequence (5' → 3′)	Length (bases)
87	TP	AGAGTCTAGACTCGTGGTGACTGCGAATTTTGG	33
	FP	AGGACGCTGAGATGCGTCCTACAGGCGGGGTTTTTCTTGTTGAC	44
	BP	TCAATTTTCTAGGGG	15
	OP	CAGGACAAGTTGGAGGAC	18
	oBP	AATCCTCACAAZACCG	16
170	TP	GATGTGTCTGCGGCGTTCCAGAAGAACCAA	30
	FP	AGGACGCTGAGATGCGTCCTGACTTCTCTCAATTTTCTAGG	41
	BP	ATCCTGCTGCTATGCCTCATCT	22
	OP	ACGGGCAACATACCTTG	17
	oBP	GCAGZCCCCAAC	12
203	TP	TTGTCCTGGCTATCGCATAGCAGCAGGATGAAGAGGA	37
	FP	GCATTCGCCCTCCAATCACTCACCAACC	28
	BP	TCTGCGGCGTTTTATC	16
	OP	TAGTCCAGAAGAACCAAC	18
	oBP	CCTGTCCZCCAAC	13
390–392	TP	CTTGAGCAGGAGTCGTGCAGGTGTCCTCTAATTCCAGG	38
	FP	GCGACTCGCTCCGAAGGTTTTGTACAGC	28
	BP	GTCCCGTGCTGGT	13
	OP	CTGCTGCTATGCCTCATC	18
	oBP-D	GGGATACAZAGAGGTT	16
	oBP	GGGAAACAZAGAGGTT	16

### SmartAmp reaction

Each SmartAmp reaction mixture was carried in a total volume of 25 µl and contained 3.2 µM FP, 3.2 µM TP, 1.2 µM BP, 0.8 µM oBP (Eprimer), 0.4 µM OP, 1.4 mM dNTPs, 20 mM Tris–HCl pH 8.0 (Sigma-Aldrich), 30 mM potassium acetate, 10 mM (NH_4_)_2_SO_4_ (Sigma-Aldrich), 8 mM MgSO_4_ (Sigma-Aldrich), 0.1% Tween 20, 2% Dextran (Wako, Takasaki, Japan), 5 µg acetylated BSA (Invitrogen, Tokyo, Japan) and 24 units of *Aac* DNA polymerase I (DNAFORM, Yokohama, Japan), and DNA sample. Samples for genotyping (12.5 µl/well) were prepared by adding 0.6 µl of 1 M NaOH to 11.9 µl of 12,500 copies of purified plasmid or viral DNA per well. The samples were heat-denatured at 95 °C for 3 min and chilled 3 min at 4 °C before adding 12.5 µl of the reaction mixture. All reactions were performed on LightCycler 480 II (Roche Diagnostics, Mannheim, Germany). Amplification was run for 60 cycles of 1 min at 67 °C and fluorescence signals were detected during each cycle using a custom thiazole orange filter range (excitation: 498 nm, emission: 580 nm). The data was transferred to Microsoft Excel (Microsoft, Redmond, VA, USA) for plotting.

### Plasmid template sequences

Eight different pEX-A2J2 vectors containing the 681 bp-long full HBV S-region genotype-specific consensus sequences (A–H) were ordered from Eurofins Genomics. A map of these plasmids can be found in Additional file 3.

### HBP25, HepG2.2.15.7 and patient serum samples

Supernatant from HBV-producing HBP25 [28] (genotype C) and HepG2.2.15.7 [29, 30] (genotype D) cell line cultures were kept at − 80 °C until use. After thawing, either 100 µl of 10^4^ copies/µl HBP25 or 15 µl of 10^7^ copies/µl HepG2.2.15.7 mixed to 85 µl of distilled water were purified using NucleoSpin^®^ Plasma XS following the manufacturer instructions (Takara Bio, Shiga, Japan) and eluted in 30 µl final volume. After purification, the samples were diluted to 10^4^ copies/ml.

Human serum samples (15 µl each, n = 9) were obtained from HBV carriers after obtaining written informed consent for the donation and evaluation of blood samples, according to The Code of Ethics of the World Medical Association (Declaration of Helsinki) and the authors’ institutional review board approval [number: Wako3 26–25(3)].The sample concentrationsof about 10^9^ copies/ml, were measured by quantitative PCR, and their genotypes, measured by PCR-Invader assay (outsourced to BML, Tokyo, Japan; assay code 03,395), are given in Additional file 4. As for the HepG2.2.15.7 sample above, 15 µl of serum mixed to 85 µl of distilled water were purified using NucleoSpin^®^ Plasma XS and eluted in 30 µl final volume. After purification, the samples were run on SmartAmp assays, with their concentration estimated to range from 10^5^ to 10^6^ copies/reaction.

### Sanger sequencing

All 9 human samples were genotyped and examined for the presence of mutations, as described previously [19]. PrimeSTAR (Takara Bio) PCR reaction was performed following the manufacturer’s instructions and using the following primers: HBV_MAFFT.Bf.41-18, CCTAGGACCCCTGCTCGT; and HBV_MAFFT.Br.601-16, ACAGACTTGGCCCCCA. Thermal cycling conditions included preincubation at 95 °C for 30 s, followed by 50 cycles at 98 °C for 10 s, 60 °C for 5 s, and 72 °C for 36 s, and extension at 72 °C for 5 min. The PCR products were purified using the QIAquick PCR purification kit (Qiagen, Tokyo, Japan) and processed for DNA sequencing using ABI PRISM BigDye Terminator version 3.1 (Applied Biosystems, Waltham, MA, USA) with the same forward or reverse primer. Sequence data were generated using the ABI PRISM 3730 DNA Analyzer (Applied Biosystems). These sequences were compared to the consensus genotypes sequences using Clustal Omega [31] and their genotypes were assessed using HBVdb online tools [25].

## Results

**Fig. 2:**
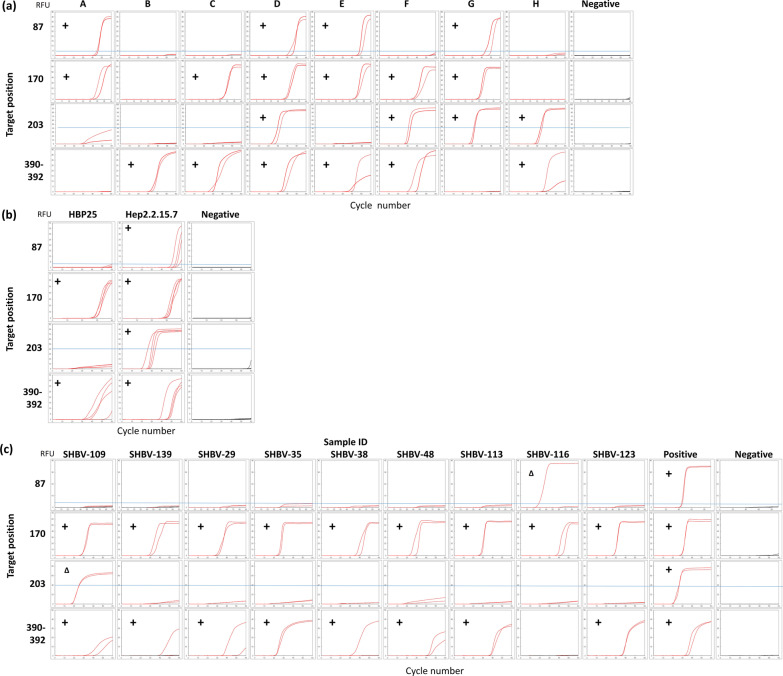
HBV genotyping detection. **a** plasmid DNA. Proof of concept using plasmid DNA of HBV full S-region, designed using each of the 8 genotypes consensus sequence (A-H), n = 2. Red sigmoid amplification curves are read as “On” positive signal and no amplification are read as “Off” negative signal. Black curves are no template negative controls (NC). **b** viral DNA from cell culture supernatant. First validation using viral DNA purified from HBP25 (genotype C) or HepG2.2.15.7 (genotype D) cell culture supernatant, n = 4. **c** Human serum samples. Nine human serum samples were genotyped with SEB Genotyping, n = 2. + : “On”, positive signal. Δ: Discordant result between the expected negative and the observed positive fluorescence signal detected

Using plasmid DNA carrying the full S-region consensus sequence for each of the 8 HBV genotypes, we tested our four genotyping primer sets targeting the positions 87, 170, 203 and 390–392 (Fig. 2a).

The expected results are a sigmoid amplification curve for a full-match between Eprimer and the template (signal On) or no amplification curve for a mismatch (signal Off). Because Eprimers bind tightly to their sequence-specific targets and a single mutation can inhibit the emission of fluorescence, we determined that even one positive amplification signal out of multiple replicates should be read as positive or “On”, and there should be no false positive, in theory. Conversely, the fluorescence signal emitted by ECHOs is very strong and can be detected even at low emission levels and there should be no false negative within the limit of detection of the assay. The combination of these On/Off signals for each of the 4 targeted positions provides a unique binary code permitting the specific genotyping of the sample tested. Comparing the amplification curve signals for each plasmid template (Fig. 2a) with the signal detection code (Table 2), we can see that each of the 8 samples were perfectly identical to the digitized On/Off pattern specific to the corresponding genotype.

**Table 2:** On/Off fluorescence signal and unique digitized binary codes

Genotype	87	170	203	390–392	Digitized
A	** + **	** + **	−	−	1100
B	−	−	−	** + **	0001
C	−	** + **	−	** + **	0101
D	** + **	** + **	** + **	** + **	1111
E	** + **	** + **	−	** + **	1101
F	−	** + **	** + **	** + **	0111
G	** + **	** + **	** + **	−	1110
H	−	−	** + **	** + **	0011

On/Off fluorescence signal emitted by Eprimer for each targeted nucleotide position (+ : On and −: Off) and the unique digitized code corresponding to each genotype

Of note, the SmartAmp primer set for target 203 (and to a lesser extent target 87) sometimes emits a low non-specific fluorescent signal. Given that the signal intensity and the amplification curves of these targets are quite different from the expected intensity and sigmoid curves, they are considered as background emissions and can easily be interpreted as noise rather than a positive signal (Fig. 2a: genotype A target 203 and genotype H target 87). Alternatively, a signal intensity cutoff can be set for an easier interpretation (cutoff at 40 RFU for target 203 and at 3 RFU for target 87). Moreover, the melting curve analysis of such non-specific signals shows a different Tm value from the true-positive samples (Additional file5), confirming that the non-specific product is clearly different from the true-positive one and thus the signal should be read as negative.

After successful genotyping of plasmid DNA, we tested our technique on natural viral DNA. The HBV25 cell line is infected with HBV genotype C [28] and HepG2.2.15.7 with genotype D [29, 30] and both cell lines release viral particles in the cell culture supernatant. The amplification curve patterns clearly follow the On/Off binary code specific to genotype C for HBP25 (1010) or genotype D for HepG2.2.15.7 (1111) (Fig. 2b). To confirm that our method was working in clinical samples, we then proceeded to test our assay on serum samples from patients chronically infected with HBV. Because the number of samples was limited and all carried the same genotype, our study cannot qualify as a clinical study and should be considered a proof of concept. Based on preliminary results on the limit of detection of our primer sets (Additional file 6), nine human serum samples (Fig. 2c) were tested at 1.25 × 10^5^ copies/reaction. The positive control was our plasmid DNA genotype D at 1.25 × 10^4^ copies/reaction. The amplification curve patterns for each sample were analyzed to determine their specific binary code and compared to the genotype given in the patient cards. The samples were also subjected to Sanger sequencing and genotyped. The concordance between the patient cards (genotyped by PCR-Invader assay) and Sanger sequencing was 100% (9/9). Using SEB Genotyping, 7 out of 9 samples (78%) had concordant results with the EIA genotyping and Sanger sequencing. One sample (SHBV-109) was ambiguous, showing the pattern “0111” for genotype F. Sequence analysis identified it as genotype C with a T > C mutation in the target position 203 inducing this pattern. Sample SHBV-116 was genotyped as A by our method, as one of the two replicates for target position 87 showed a clear amplification signal, but as C by both the reference method and Sanger sequencing. Further post-amplification testing using melting curve showed the melting peak patterns were clearly different between this specific case and the positive control (Additional file 7) or other genotypes (Additional file 5). The amplification product obtained is not the same as for other samples and a melting curve analysis could distinctly identify a false positive.

## Discussion

Recent international guidelines [32] for treatment of HBV patients recommend HBV genotyping before initiating pegylated interferon therapy, making it necessary to develop new, simple, convenient and accurate technologies for HBV genotyping even at community hospitals and clinics. Our new technique’s one-well amplification and detection step reduces the risk of contamination compared to others (e.g. PCR invader assay), and only 4 regions need to be detected (15 for the oligonucleotides microarray [33]). A digital binary code for HBV detection was developed using lateral flow immunoassay [34], but it required an 8-digits binary code to distinguish between genotypes A-B-C and D, while our 4-digits binary code can distinguish 8 HBV genotypes from A to H.

We showed that our technology works well on plasmid DNA and on viral particles extracted from cell culture supernatant. One limitation in our experiments was the very small number (n = 9) and very low amount of patient serum samples (15 µl each) available, all of them being genotype C. Indeed, all samples are from Japanese patients with chronic HBV infection and in this population around 80% are infected with genotype C [35–37]. The number is also low because we tested human samples as an example to confirm whether the expected theoretical pattern was obtained with our method using actual samples rather than to extensively validate it in a clinical study. While 7 out of 9 samples did show the expected pattern, two samples had conflicting results. Long-term storage of low amount of sample as well as the DNA extraction step increase the risk of degradation and loss of template [38], which may have caused the discordant results found in sample SHBV-116 or a rare event of primer dimerization induced this signal, as this pattern was only seen in one replicate of one patient sample while all other patient samples as well as plasmids and infected cell lines showed the expected amplification pattern. While this specific sample is not representative of the assay, its false positive result highlights that our study needs further optimization and testing at a larger scale before aiming for the clinical setting. Another limitation of nucleic acid detection-based methods is the vulnerability of the signal to mutations. We have selected the 4 target positions to be at least 90% conserved within one genotype while different from other genotypes, or otherwise noted in Fig. 1c. Target position 203 is 86% conserved as T in genotype C but one of our samples (SHBV-109) was sequenced as carrying a minor variant having a T > C mutation in this position resulting in a miscalling as genotype F.While it is unfortunate to have a minor variant in such a low number of samples, the issue here does not lay with the SEB Genotyping method but with the careful selection of the target, pointing out that the target 203 may not be an adequate position for our purpose. It may be necessary for future users selecting their own SEB Genotyping targets to revise the cut-off to higher than 90% to avoid such discordant results. Regarding the low non-specific background signal that is sometimes observed, one could design Eprimers with the labelled base within 3 nucleotides of the targeted variant, anywhere on the Eprimer sequence and not only at the penultimate 3′-end nucleotide, interfering with the binding of the Eprimer to the viral DNA, preventing the quenched thiazole orange moieties from separating from each other and effectively inhibiting the emission of fluorescence in case of mismatch.

We had no information about the past treatment regimen of the 9 patient samples tested in this study or about their drug resistance status. Because the selected targets are highly conserved within each genotype, we believe that they are somehow necessary for the virus life cycle and the accumulation of mutations due to resistance to treatment would probably not influence these specific sites, although this point should be further studied and verified. If the DNA of HBV can be amplified from the patient serum (such as patients with persistent viremia or virological breakthrough), regardless of past treatment, SEB Genotyping can be used. To our knowledge, current treatments or those in development (nucleot/side analogs, siRNA, various inhibitors of the viral cycle or immunomodulators) [39] do not directly induce mutations in the HBV genome and should not have any effect on the detection and genotyping of the virus by SEB Genotyping. A hypothetical therapy using CRISPR or other similar technology to insert a mutation specifically in the regions targeted in our manuscript would prevent SEB Genotyping from being used in its current form, but it would be possible to design new primers to target another region to solve the issue. From our point of view, only the viral load and detection limit would determine whether or not SEB Genotyping can be used to genotype patients for prediction of treatment resistance.

## Conclusion

We developed a new usage for SmartAmp-Eprimer, making use of the strongly sequence-specific binding of Eprimers to their targets to create an On/Off genotyping tool specifically designed for highly variable targets. Regarding the application of SEB Genotyping to the detection of HBV genotypes, while the human serum sample testing results shown here permits us to foresee satisfying genotyping results with our new method, they are only a proof of concept and further testing on a larger number of samples as well as human sera with other HBV genotypes will be necessary for a better representation of the sensibility and accuracy of our assay in clinical settings.

Future development of SEB Genotyping would include the expansion to other targets to detect between various bacterial or viral infections, for example for the fast, on-site distinction between the crucial variants of concern of the SARS-CoV-2 virus [40–42] in COVID-19 patients; signature mutations linked to cancer development and prognosis or any other highly variable part of a genome of interest. SEB Genotyping was optimized so that all 4 primer sets run under the same conditions, reducing the technical hurdles, and could easily be adapted into a microfluidics chip.

## Supplementary Information

**Additional file 1**: Consensus sequences and PID results. Consensus sequences for each genotype (A–H) and the pan-genotype, and corresponding PID as calculated by MAFFT.

**Additional file 2**: Consensus sequence alignments results from various tools. We compared the alignment results of Kalign, MAFFT (8 consensus sequences) and Jalview/MAFFT (over 20,000 sequences). Aligning the 8 HBV genotypes before creating a consensus avoided bias towards the more common genotypes (C and B) and excluded the sequences with long insertions (highlighted in green).

**Additional file 3:** Plasmid sequences. A map of the pEX-A2J2 vector into which were inserted one by one each of the 8 consensus sequences (A-H) described in Additional file 1.

**Additional file 4**: Human serum samples. Human serum samples, anonymized, genotyped with EIA and the concentration of HBV DNA detected by qPCR at the time of registration.

**Additional file 5**: HBV genotyping Amplification and Melting curve analysis of non-specific fluorescent signals. Test on plasmid DNA (full S-region, designed using each of the 8 genotypes consensus sequence, n = 2). Red melting curves show a sharp positive signal peak when the correct product is amplified and the Eprimer-oBP binds to its target (target 87 genotype A or target 203 genotype F) while non-specific fluorescent signal, below the threshold (blue line) and with a non-sigmoid amplification curve, show a flat melting curve (target 87 genotype F or target 203 genotype A). Black curves are no template negative controls (NC). If amplification curves are hard to interpret, the melting curves can help decide if a signal should be considered positive or negative.

**Additional file 6**: Preliminary results on limit of detection. We tested various reaction volumes and template concentrations to roughly determine the detection limit of our assays. These results are not exhaustive and do not follow the exact same protocol as used in the main body of our manuscript and should be considered preliminary.

**Additional file 7**: Melting curves analysis for target position 87, sample SHBV-116. Test on human serum sample SHBV-116, (n = 2). Red melting curves show a sharp positive signal double-peak when the correct product is amplified and the Eprimer-oBP binds to its target as is shown in the positive control genotype D (PC-D) while non-specific fluorescent signal shows a strikingly differently shaped melting curve in SHBV-116. Black curves are no template negative controls (NC). If amplification curves are hard to interpret, the melting curves can help decide if a signal should be considered positive or negative.

## Data Availability

All data generated or analyzed during this study are included in this published article [and its Additional files] or have been deposited in the European Nucleotide Archive (ENA) at EMBL-EBI under accession number PRJEB51673 (https://www.ebi.ac.uk/ena/browser/view/PRJEB51673).

## Abbreviations

HBV: Hepatitis B virus
PCR: Polymerase chain reaction
ECHO: Exciton-controlled hybridization-sensitive fluorescent oligonucleotides
SEB Genotyping: SmartAmp-Eprimer Binary code genotyping

## References

[1] Kreimer AR, Clifford GM, Boyle P, Franceschi S (2005). Human papillomavirus types in head and neck squamous cell carcinomas worldwide: a systemic review. Cancer Epidemiol Biomark Prevent Am Assoc Cancer Res..

[2] Viens LJ, Henley SJ, Watson M, Markowitz LE, Thomas CC, Thompson TD (2016). Human papillomavirus-associated cancers—United States, 2008–2012. MMWR Morb Mortal Wkly Rep.

[3] World Health Organization. Global Hepatitis Report 2017. Geneva: World Health Organization; 2017 Licence: CC BY-NC-SA 3.0 IGO. WHO. 2017.

[4] Velkov S, Ott JJ, Protzer U, Michler T (2018). The global hepatitis B virus genotype distribution approximated from available genotyping data. Genes (Basel)..

[5] Guirgis BSS, Abbas RO, Azzazy HME (2010). Hepatitis B virus genotyping: current methods and clinical implications. Int J Infect Dis.

[6] Rajoriya N, Combet C, Zoulim F, Janssen HLA (2017). How viral genetic variants and genotypes influence disease and treatment outcome of chronic hepatitis B. Time for an individualised approach?. J Hepatol.

[7] Wan YM, Li YH, Xu ZY, Wu HM, Wu XN, Xu Y (2018). Genotype matters in patients with acute-on-chronic liver failure due to reactivation of chronic hepatitis B. Clin Transl Gastroenterol.

[8] Munkongdee T, Chen P, Winichagoon P, Fucharoen S, Paiboonsukwong K (2020). Update in laboratory diagnosis of thalassemia. Front Mol Biosci.

[9] Chan HT, Chin YM, Low S-K (2019). The roles of common variation and somatic mutation in cancer pharmacogenomics. Oncol Ther.

[10] Mbanefo A, Kumar N (2020). Evaluation of malaria diagnostic methods as a key for successful control and elimination programs. Trop Med Infect Dis.

[11] James AS, Alwneh JI (2020). COVID-19 infection diagnosis: potential impact of isothermal amplification technology to reduce community transmission of SARS-CoV-2. Diagnostics.

[12] Luo Z, Ang MJY, Chan SY, Yi Z, Goh YY, Yan S (2020). Combating the coronavirus pandemic: early detection, medical treatment, and a concerted effort by the global community. Research.

[13] Abduljalil JM (2020). Laboratory diagnosis of SARS-CoV-2: available approaches and limitations. N Microb N Infect..

[14] Mitani Y, Lezhava A, Kawai Y, Kikuchi T, Oguchi-Katayama A, Kogo Y (2007). Rapid SNP diagnostics using asymmetric isothermal amplification and a new mismatch-suppression technology. Nat Methods.

[15] Ikeda S, Okamoto A (2008). Hybridization-sensitive On–Off DNA probe: application of the exciton coupling effect to effective fluorescence quenching. Chem An Asian J..

[16] Okamoto A (2010). Excitonic interaction: another photophysical process for fluorescence-controlled nucleic acid sensing. Chem Rec.

[17] Kimura Y, Hanami T, Tanaka Y, De Hoon MJL, Soma T, Harbers M (2012). Effect of thiazole orange doubly labeled thymidine on DNA duplex formation. Biochemistry.

[18] Hanami T, Delobel D, Kanamori H, Tanaka Y, Kimura Y, Nakasone A (2013). Eprobe mediated real-time PCR monitoring and melting curve analysis. PLoS ONE.

[19] Atsumi J, Hanami T, Enokida Y, Ogawa H, Delobel D, Mitani Y (2015). Eprobe-mediated screening system for somatic mutations in the KRAS locus. Oncol Rep.

[20] Lezhava A, Ishidao T, Ishizu Y, Naito K, Hanami T, Katayama A (2010). Exciton primer-mediated SNP detection in SmartAmp2 reactions. Hum Mutat.

[21] Enokida Y, Shimizu K, Atsumi J, Lezhava A, Tanaka Y, Kimura Y (2013). Rapid Detection of SNP (c.309T>G) in the MDM2 Gene by the Duplex SmartAmp Method. PLoS ONE.

[22] Enokida Y, Shimizu K, Kakegawa S, Atsumi J, Takase Y, Miyamae Y (2014). Single-nucleotide polymorphism (c.309T>G) in the MDM2 gene and lung cancer risk. Biomed Rep..

[23] Enokida Y, Shimizu K, Atsumi J, Kakegawa S, Takase Y, Kaira K, et al. Prognostic potential of the MDM2 309T>G polymorphism in stage I lung adenocarcinoma. Cancer Med. 2016. 10.1002/cam4.750 PMC4884639 27228500

[24] Kawai Y, Kimura Y, Lezhava A, Kanamori H, Usui K, Hanami T (2012). One-step detection of the 2009 pandemic influenza a(H1N1) virus by the RT-smartamp assay and its clinical validation. PLoS ONE.

[25] Hayer J, Jadeau F, Deléage G, Kay A, Zoulim F, Combet C (2013). HBVdb: a knowledge database for Hepatitis B Virus. Nucleic Acids Res.

[26] Waterhouse AM, Procter JB, Martin DMA, Clamp M, Barton GJ (2009). Jalview Version 2—A multiple sequence alignment editor and analysis workbench. Bioinformatics.

[27] Kimura Y, Soma T, Kasahara N, Delobel D, Hanami T, Tanaka Y (2016). Edesign: Primer and enhanced internal probe design tool for quantitative PCR experiments and genotyping assays. PLoS ONE.

[28] Tsuge M, Hiraga N, Zhang Y, Yamashita M, Sato O, Oka N (2018). Endoplasmic reticulum-mediated induction of interleukin-8 occurs by hepatitis B virus infection and contributes to suppression of interferon responsiveness in human hepatocytes. Virology.

[29] Ogura N, Watashi K, Noguchi T, Wakita T (2014). Formation of covalently closed circular DNA in Hep38.7-Tet cells, a tetracycline inducible hepatitis B virus expression cell line. Biochem Biophys Res Commun.

[30] Sells MA, Chen ML, Acs G (1987). Production of hepatitis B virus particles in Hep G2 cells transfected with cloned hepatitis B virus DNA. Proc Natl Acad Sci USA.

[31] Madeira F, Park YM, Lee J, Buso N, Gur T, Madhusoodanan N (2019). The EMBL-EBI search and sequence analysis tools APIs in 2019. Nucleic Acids Res.

[32] Terrault NA, Lok ASF, Mcmahon BJ, Chang K-M, Hwang JP, Jonas MM (2018). Update on prevention, diagnosis, and treatment of chronic hepatitis B: AASLD 2018 hepatitis B Guidance American Association for the study of liver diseases. Hepatology.

[33] Song Y, Dai E, Wang J, Liu H, Zhai J, Chen C (2006). Genotyping of hepatitis B virus (HBV) by oligonucleotides microarray. Mol Cell Probes.

[34] Qiu X, Song L, Yang S, Guo M, Yuan Q, Ge S (2016). A fast and low-cost genotyping method for hepatitis B virus based on pattern recognition in point-of-care settings. Sci Rep.

[35] Matsuura K, Tanaka Y, Hige S, Yamada G, Murawaki Y, Komatsu M (2009). Distribution of hepatitis B virus genotypes among patients with chronic infection in Japan shifting toward an increase of genotype A. J Clin Microbiol.

[36] Uchida Y, Nakao M, Yamada S, Tsuji S, Uemura H, Kouyama J, et al. Superiority of tenofovir alafenamide fumarate over entecavir for serum HBsAg level reduction in patients with chronic HBV infection: a 144-week outcome study after switching of the nucleos(t)ide analog. Huang J-F, editor. PLoS One. 2022;17(2):e0262764. 10.1371/journal.pone.0262764 PMC8856517 35180213

[37] Orito E, Mizokami M (2003). Hepatitis B virus genotypes and hepatocellular carcinoma in Japan. Intervirology.

[38] Sozzi G, Roz L, Conte D, Mariani L, Andriani F, Verderio P (2005). Effects of prolonged storage of whole plasma or isolated plasma DNA on the results of circulating DNA quantification assays. J Natl Cancer Inst.

[39] Spyrou E, Smith CI, Ghany MG (2020). Hepatitis B: Current Status of Therapy and Future Therapies. Gastroenterol Clin North Am.

[40] Singh J, Samal J, Kumar V, Sharma J, Agrawal U, Ehtesham NZ (2021). Structure–function analyses of new SARS-CoV-2 Variants B.1.1.7, B.1.351 and B.1.1.28.1: clinical, diagnostic, therapeutic and public health implications. Viruses.

[41] Davies NG, Abbott S, Barnard RC, Jarvis CI, Kucharski AJ, Munday JD (2021). Estimated transmissibility and impact of SARS-CoV-2 lineage B.1.1.7 in England. Science (80-)..

[42] Tegally H, Wilkinson E, Giovanetti M, Iranzadeh A, Fonseca V, Giandhari J (2021). Detection of a SARS-CoV-2 variant of concern in South Africa. Nature.

