# Supplementary material for: SEB genotyping: SmartAmp-Eprimer binary code genotyping for complex, highly variable targets applied to HBV
Source: BMC Infect Dis. 2022 Jun 3;22:516. doi: 10.1186/s12879-022-07458-4 (PMC9164387; doi:10.1186/s12879-022-07458-4)
Supplement: Supplementary file 3 — Additional file 3: Plasmid sequences. A map of the pEX-A2J2 vector into which were inserted one by one each of the 8 consensus sequences (A-H) described in Additional file 1. [file 12879_2022_7458_MOESM3_ESM.pdf]

pEX-A2J2 vector map and full sequence

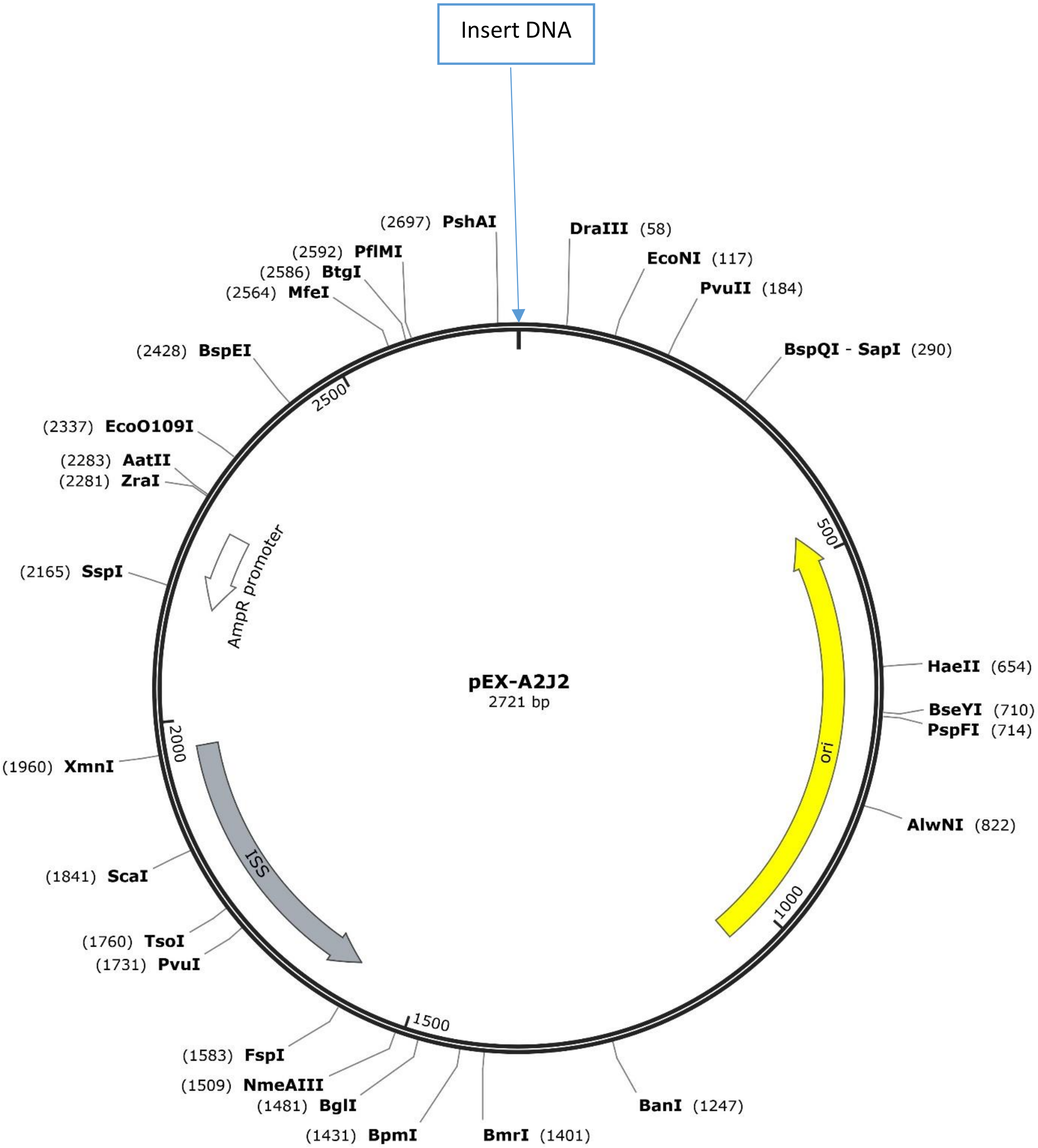

Sequence

<< Insert DNA >>
AGTCAGTGGATACGCCAAATGCCTTCATGCGCCCAAAACATGTTGGCATGTTCACGATGTGCCCAACGATGTCCTT
CCCAACGTGCTCTACCGGCATGATCAAAGCTATGCGCCTTAAACAGGTCATGCGCTGAATAGTGGCGTTCGATTGC
CCAACCGTAGAAGTCTTTGCCTGTCGTGCCAGCTGCATTAATGAATCGGCCAACGCGCGGGGAGAGGCGGTTTGC
GTATTGGGCAGAGAATATAAAAAGCCAGATTATTAATCCGGCTTTTTTATTATTTGCTCTTCCGCTTCCTCGCTCACT
GACTCGCTGCGCTCGGTCGTTCGGCTGCGGCGAGCGGTATCAGCTCACTCAAAGGCGGTAATACGGTTATCCACA
GAATCAGGGGATAACGCAGGAAAGAACATGTGAGCAAAAGGCCAGCAAAAGGCCAGGAACCGTAAAAAGGCCG
CGTTGCTGGCGTTTTTCCATAGGCTCCGCCCCCCTGACGAGCATCACAAAAATCGACGCTCAAGTCAGAGGTGGCG
AAACCCGACAGGACTATAAAGATACCAGGCGTTTCCCCCTGGAAGCTCCCTCGTGCGCTCTCCTGTTCCGACCCTG
CCGCTTACCGGATACCTGTCCGCCTTTCTCCCTTCGGGAAGCGTGGCGCTTTCTCATAGCTCACGCTGTAGGTATCT
CAGTTCGGTGTAGGTCGTTCGCTCCAAGCTGGGCTGTGTGCACGAACCCCCCGTTCAGCCCGACCGCTGCGCCTTA
TCCGGTAACTATCGTCTTGAGTCCAACCCGGTAAGACACGACTTATCGCCACTGGCAGCAGCCACTGGTAACAGGA
TTAGCAGAGCGAGGTATGTAGGCGGTGCTACAGAGTTCTTGAAGTGGTGGCCTAACTACGGCTACACTAGAAGAA
CAGTATTTGGTATCTGCGCTCTGCTGAAGCCAGTTACCTTCGGAAAAAGAGTTGGTAGCTCTTGATCCGGCAAACA
AACCACCGCTGGTAGCGGTGGTTTTTTTGTTTGCAAGCAGCAGATTACGCGCAGAAAAAAAGGATCTCAAGAAGA
TCCTTTGATCTTTTCTACGGGGTCTGACGCTCAGTGGAACGAAAACTCACGTTAAGGGATTTTGGTCATGAGATTAT
CAAAAAGGATCTTCACCTAGATCCTTTTAAATTAAAAATGAAGTTTTAAATCAATCTAAAGTATATATGAGTAAACT
TGGTCTGACAGTTACCAATGCTTAATCAGTGAGGCACCTATCTCAGCGATCTGTCTATTTCGTTCATCCATAGTTGC
CTGACTCCCSncngrmrGCTTTGATCATGCCGGTAGSncngrmrRTTTGCGCTCAGCCCATSncnsrtDNACGTCGTGTA
GATAACTACGATACGGGAGGGCTTACCATCTGGCCCCAGTGCTGCAATGATACCGCGACTCCCACGCTCACCGGCT
CCAGATTTATCAGCAATAAACCAGCCAGCCGGAAGGGCCGAGCGCAGAAGTGGTCCTGCAACTTTATCCGCCTCC
ATCCAGTCTATTAATTGTTGCCGGGAAGCTAGAGTAAGTAGTTCGCCAGTTAATAGTTTGCGCAACGTTGTTGCCA
TTGCTACAGGCATCGTGGTGTCACGCTCGTCGTTTGGTATGGCTTCATTCAGCTCCGGTTCCCAACGATCAAGGCG
AGTTACATGATCCCCCATGTTGTGCAAAAAAGCGGTTAGCTCCTTCGGTCCTCCGATCGTTGTCAGAAGTAAGTTG
GCCGCAGTGTTATCACTCATGGTTATGGCAGCACTGCATAATTCTCTTACTGTCATGCCATCCGTAAGATGCTTTTCT
GTGACTGGTGAGTACTCAACCAAGTCATTCTGAGAATAGTGTATGCGGCGACCGAGTTGCTCTTGCCCGGCGTCA
ATACGGGATAATACCGCGCCACATAGCAGAACTTTAAAAGTGCTCATCATTGGAAAACGTTCTTCGGGGCGAAAA
CTCTCAAGGATCTTACCGCTGTTGAGATCCAGTTCGATGTAACCCACTCGTGCACCCAACTGATCTTCAGCATCTTTT
ACTTTCACCAGCGTTTCTGGGTGAGCAAAAACAGGAAGGCAAAATGCCGCAAAAAAGGGAATAAGGGCGACACG
GAAATGTTGAATACTCATACTCTTCCTTTTTCAATATTATTGAAGCATTTATCAGGGTTATTGTCTCATGAGCGGATA
CATATTTGAATGTATTTAGAAAAATAAACAAATAGGGGTTCCGCGCACATTTCCCCGAAAAGTGCCACCTGACGTC
TAAGAAACCATTATTATCATGACATTAACCTATAAAAATAGGCGTATCACGAGGCCCTTTCGTCTCGCGCGTTTCGG
TGATGACGGTGAAAACCTCTGACACATGCAGCTCAAAAAAAGGATCTCAAGAAGATCCTTTGATTTTCCGGAGAC
GGTCACAGCTTGTCTGTAAGCGGATGCCGGGAGCAGACAAGCCCGTCAGGGCGCGTCAGCGGGTGTTGGCGGGT
GTCGGGGCTGGCTTAACTATGCGGCATCAGAGCAGATTGTACTGAGAGTGCACCAATTGAAAAGTAGTGTCGTTC
CCACGGAATGGCATAGGTCAGCCAGATACGTTCGGCATTTTTGCGCTCAGCCCATTTGTGGAAGATGCGTTCCCAA
TGACCTTCCGCAGATAAGCGGGTAAAATCCCGACTATGGTCGCTTTTGTGCTCACGGCAT

In the “Insert DNA” frame was inserted one of the eight following 681bp sequences, a consensus sequence of one of the 8 genotypes A to H of the HBV virus full S-region:

>HBV_MAFFT_consensus_A
ATGGAGAACATCACATCAGGATTCCTAGGACCCCTGCTCGTGTTACAGGCGGGGTTTTTCTTGTTGACAAGAATCC
TCACAATACCGCAGAGTCTAGACTCGTGGTGGACTTCTCTCAATTTTCTAGGGGGATCACCCGTGTGTCTTGGCCA
AAATTCGCAGTCCCCAACCTCCAATCACTCACCAACCTCCTGTCCTCCAATTTGTCCTGGTTATCGCTGGATGTGTCT
GCGGCGTTTTATCATATTCCTCTTCATCCTGCTGCTATGCCTCATCTTCTTATTGGTTCTTCTGGATTATCAAGGTATG
TTGCCCGTTTGTCCTCTAATTCCAGGATCAACAACAACCAGTACGGGACCATGCAAAACCTGCACGACTCCTGCTCA
AGGCAACTCTATGTTTCCCTCATGTTGCTGTACAAAACCTACGGATGGAAATTGCACCTGTATTCCCATCCCATCGT
CCTGGGCTTTCGCAAAATACCTATGGGAGTGGGCCTCAGTCCGTTTCTCTTGGCTCAGTTTACTAGTGCCATTTGTT
CAGTGGTTCGTAGGGCTTTCCCCCACTGTTTGGCTTTCAGCTATATGGATGATGTGGTATTGGGGGCCAAGTCTGT
ACAGCATCGTGAGTCCCTTTATACCGCTGTTACCAATTTTCTTTTGTCTCTGGGTATACATTTAA

>HBV_MAFFT_consensus_B
ATGGAGAACATCGCATCAGGACTCCTAGGACCCCTGCTCGTGTTACAGGCGGGGTTTTTCTTGTTGACAAAAATCC
TCACAATACCACAGAGTCTAGACTCGTGGTGGACTTCTCTCAATTTTCTAGGGGGAACACCCGTGTGTCTTGGCCA
AAATTCGCAGTCCCAAATCTCCAGTCACTCACCAACCTGTTGTCCTCCAATTTGTCCTGGTTATCGCTGGATGTGTCT
GCGGCGTTTTATCATCTTCCTCTGCATCCTGCTGCTATGCCTCATCTTCTTGTTGGTTCTTCTGGACTATCAAGGTAT
GTTGCCCGTTTGTCCTCTAATTCCAGGATCATCAACAACCAGCACCGGACCATGCAAAACCTGCACAACTCCTGCTC
AAGGAACCTCTATGTTTCCCTCATGTTGCTGTACAAAACCTACGGACGGAAACTGCACCTGTATTCCCATCCCATCA
TCTTGGGCTTTCGCAAAATACCTATGGGAGTGGGCCTCAGTCCGTTTCTCTTGGCTCAGTTTACTAGTGCCATTTGT
TCAGTGGTTCGTAGGGCTTTCCCCCACTGTCTGGCTTTCAGTTATATGGATGATGTGGTTTTGGGGGCCAAGTCTG
TACAACATCTTGAGTCCCTTTATGCCGCTGTTACCAATTTTCTTTTGTCTTTGGGTATACATTTAA

>HBV_MAFFT_consensus_C
ATGGAGAACACAACATCAGGATTCCTAGGACCCCTGCTCGTGTTACAGGCGGGGTTTTTCTTGTTGACAAGAATCC
TCACAATACCACAGAGTCTAGACTCGTGGTGGACTTCTCTCAATTTTCTAGGGGGAGCACCCACGTGTCCTGGCCA
AAATTCGCAGTCCCCAACCTCCAATCACTCACCAACCTCTTGTCCTCCAATTTGTCCTGGCTATCGCTGGATGTGTCT
GCGGCGTTTTATCATATTCCTCTTCATCCTGCTGCTATGCCTCATCTTCTTGTTGGTTCTTCTGGACTACCAAGGTAT
GTTGCCCGTTTGTCCTCTACTTCCAGGAACATCAACTACCAGCACGGGACCATGCAAGACCTGCACGATTCCTGCTC
AAGGAACCTCTATGTTTCCCTCTTGTTGCTGTACAAAACCTTCGGACGGAAACTGCACTTGTATTCCCATCCCATCAT
CCTGGGCTTTCGCAAGATTCCTATGGGAGTGGGCCTCAGTCCGTTTCTCCTGGCTCAGTTTACTAGTGCCATTTGTT
CAGTGGTTCGTAGGGCTTTCCCCCACTGTTTGGCTTTCAGTTATATGGATGATGTGGTATTGGGGGCCAAGTCTGT
ACAACATCTTGAGTCCCTTTTTACCTCTATTACCAATTTTCTTTTGTCTTTGGGTATACATTTGA

>HBV_MAFFT_consensus_D
ATGGAGAACATCACATCAGGATTCCTAGGACCCCTGCTCGTGTTACAGGCGGGGTTTTTCTTGTTGACAAGAATCC
TCACAATACCGCAGAGTCTAGACTCGTGGTGGACTTCTCTCAATTTTCTAGGGGGAACTACCGTGTGTCTTGGCCA
AAATTCGCAGTCCCCAACCTCCAATCACTCACCAACCTCCTGTCCTCCAACTTGTCCTGGTTATCGCTGGATGTGTCT
GCGGCGTTTTATCATCTTCCTCTTCATCCTGCTGCTATGCCTCATCTTCTTGTTGGTTCTTCTGGACTATCAAGGTATG
TTGCCCGTTTGTCCTCTAATTCCAGGATCTTCAACCACCAGCACGGGACCATGCAGAACCTGCACGACTCCTGCTCA
AGGAACCTCTATGTATCCCTCCTGTTGCTGTACCAAACCTTCGGACGGAAATTGCACCTGTATTCCCATCCCATCAT
CCTGGGCTTTCGGAAAATTCCTATGGGAGTGGGCCTCAGCCCGTTTCTCCTGGCTCAGTTTACTAGTGCCATTTGTT
CAGTGGTTCGTAGGGCTTTCCCCCACTGTTTGGCTTTCAGTTATATGGATGATGTGGTATTGGGGGCCAAGTCTGT
ACAGCATCTTGAGTCCCTTTTTACCGCTGTTACCAATTTTCTTTTGTCTTTGGGTATACATTTAA

>HBV_MAFFT_consensus_E
ATGGAAAGCATCACATCAGGATTCCTAGGACCCCTGCTCGTGTTACAGGCGGGGTTTTTCTTGTTGACAAAAATCC
TCACAATACCGCAGAGTCTAGACTCGTGGTGGACTTCTCTCAATTTTCTAGGGGGAGCTCCCGTGTGTCTTGGCCA
AAATTCGCAGTCCCCAACCTCCAATCACTCACCAACCTCTTGTCCTCCAATTTGTCCTGGCTATCGCTGGATGTGTCT
GCGGCGTTTTATCATCTTCCTCTTCATCCTGCTGCTATGCCTCATCTTCTTGTTGGTTCTTCTGGACTATCAAGGTATG
TTGCCCGTTTGTCCTCTAATTCCAGGATCATCAACCACCAGTACGGGACCCTGCCGAACCTGCACGACTCTTGCTCA
AGGAACCTCTATGTTTCCCTCATGTTGCTGTTCAAAACCTTCGGACGGAAATTGCACTTGTATTCCCATCCCATCATC
ATGGGCTTTCGGAAAATTCCTATGGGAGTGGGCCTCAGCCCGTTTCTCCTGGCTCAGTTTACTAGTGCCATTTGTTC
AGTGGTTCGCCGGGCTTTCCCCCACTGTCTGGCTTTCAGTTATATGGATGATGTGGTATTGGGGGCCAAGTCTGTA
CAACATCTTGAGTCCCTTTATACCTCTGTTACCAATTTTCTTTTGTCTTTGGGTATACATTTAA

>HBV_MAFFT_consensus_F
ATGGACAACATCACATCAGGACTCCTAGGACCCCTGCTCGTGTTACAGGCGGTGTGTTTCTTGTTGACAAAAATCC
TCACAATACCACAGAGTCTAGACTCGTGGTGGACTTCTCTCAATTTTCTAGGGGGACTACCCGGGTGTCCTGGCCA
AAATTCGCAGTCCCCAACCTCCAATCACTTACCAACCTCCTGTCCTCCAACTTGTCCTGGCTATCGTTGGATGTGTCT
GCGGCGTTTTATCATCTTCCTCTTCATCCTGCTGCTATGCCTCATCTTCTTGTTGGTTCTTCTGGACTATCAAGGTATG
TTGCCCGTTTGTCCTCTACTTCCAGGATCCACGACCACCAGCACGGGACCATGCAAAACCTGCACAACTCTTGCTCA
AGGAACCTCTATGTTTCCCTCCTGTTGCTGTTCCAAACCCTCGGACGGAAACTGCACTTGTATTCCCATCCCATCATC
TTGGGCTTTAGGAAAATACCTATGGGAGTGGGCCTCAGCCCGTTTCTCCTGGCTCAGTTTACTAGTGCAATTTGTTC
AGTGGTGCGTAGGGCTTTCCCCCACTGTCTGGCTTTTAGTTATATGGATGATCTGGTATTGGGGGCCAAATCTGTG
CAGCATCTTGAGTCCCTTTATACCGCTGTTACCAATTTTCTGTTATCTGTGGGTATCCATTTAA

>HBV_MAFFT_consensus_G
ATGGAGAACATCACATCAGGATTCCTAGGACCCCTGCTCGTGTTACAGGCGGGGTTTTTCTTGTTGACAAGAATCC
TCACAATACCGCAGAGTCTAGACTCGTGGTGGACTTCTCTCAATTTTCTAGGGGGAGTGCCCGTGTGTCCTGGCCT
AAATTCGCAGTCCCCAACCTCCAATCACTCACCAATCTCCTGTCCTCCAACTTGTCCTGGCTATCGCTGGATGTGTCT
GCGGCGTTTTATCATATTCCTCTTCATCCTGCTGCTATGCCTCATCTTCTTGTTGGTTCTTCTGGACTATCAAGGTAT
GTTGCCCGTTTGTCCTCTGATTCCAGGATCCTCGACCACCAGTACGGGACCCTGCAAAACCTGCACGACTCCTGCTC
AAGGCAACTCTATGTATCCCTCATGTTGCTGTACAAAACCTTCGGACGGAAATTGCACCTGTATTCCCATCCCATCA
TCTTGGGCTTTCGCAAAATACCTATGGGAGTGGGCCTCAGTCCGTTTCTCTTGGCTCAGTTTACTAGTGCCATTTGT
TCAGTGGTTCGTAGGGCTTTCCCCCACTGTCTGGCTTTCAGCTATATGGATGATGTGGTATTGGGGGCCAAATCTG
TACAACATCTTGAGTCCCTTTATACCGCTGTTACCAATTTTCTTTTGTCTTTGGGTATACATCTAA

>HBV_MAFFT_consensus_H
ATGGAGAACATCACATCAGGACTCCTAGGACCCCTTCTCGTGTTACAGGCGGTGTGTTTCTTGTTGACAAAAATCC
TCACAATACCACAGAGTCTAGACTCGTGGTGGACTTCTCTCAATTTTCTAGGGGTACCACCCGGGTGTCCTGGCCA
AAATTCGCAGTCCCCAATCTCCAATCACTTACCAACCTCCTGTCCTCCAACTTGTCCTGGCTATCGTTGGATGTGTCT
GCGGCGTTTTATCATCTTCCTCTTCATCCTGCTGCTATGCCTCATCTTCTTGTTGGTTCTTCTGGACTATCAAGGTATG
TTGCCCGTGTGTCCTCTACTTCCAGGATCTACAACCACCAGCACGGGACCCTGCAAAACCTGCACCACTCTTGCTCA
AGGAACCTCTATGTTTCCCTCCTGCTGCTGTACCAAACCTTCGGACGGAAATTGCACCTGTATTCCCATCCCATCATC
TTGGGCTTTCGGAAAATACCTATGGGAGTGGGCCTCAGCCCGTTTCTCTTGGCTCAGTTTACTAGTGCAATTTGTTC
AGTGGTGCGTAGGGCTTTCCCCCACTGTCTGGCTTTTAGTTATATGGATGATTTGGTATTGGGGGCCAAATCTGTG
CAGCATCTTGAGTCCCTTTATACCGCTGTTACCAATTTTTTGTTATCTGTGGGCATCCATTTGA
