# Supplementary material for: SEB genotyping: SmartAmp-Eprimer binary code genotyping for complex, highly variable targets applied to HBV
Source: BMC Infect Dis. 2022 Jun 3;22:516. doi: 10.1186/s12879-022-07458-4 (PMC9164387; doi:10.1186/s12879-022-07458-4)
Supplement: Supplementary file 4 — Additional file 4: Human serum samples. Human serum samples, anonymized, genotyped with EIA and the concentration of HBV DNA detected by qPCR at the time of registration. [file 12879_2022_7458_MOESM4_ESM.pdf]

Anonymization number	HBV DNA in serum (Log copy /mL)	GENOTYPE	HBs antigen levels (IU/mL) (CLIA)
SHBV-109	9.1	C	34,600
SHBV-139	9.1	C	19,600
SHBV-029	9	C	3,750
SHBV-035	9	C	279,000
SHBV-038	9	C	55,500
SHBV-048	9	C	229,000
SHBV-113	9	C	107,000
SHBV-116	9	C	2,200
SHBV-123	9	C	48,900
