# Supplementary figures and images for: SEB genotyping: SmartAmp-Eprimer binary code genotyping for complex, highly variable targets applied to HBV
Source: BMC Infect Dis. 2022 Jun 3;22:516. doi: 10.1186/s12879-022-07458-4 (PMC9164387; doi:10.1186/s12879-022-07458-4)

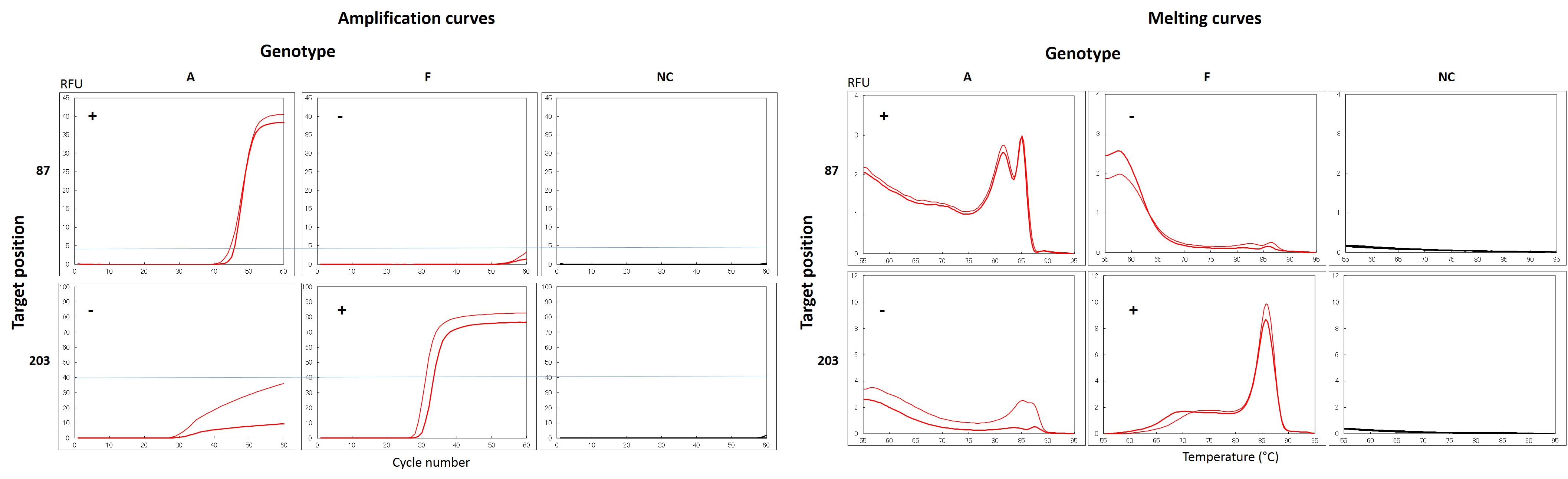

Supplement: Supplementary file 5 — Additional file 5: HBV genotyping Amplification and Melting curve analysis of non-specific fluorescent signals. Test on plasmid DNA (full S-region, designed using each of the 8 genotypes consensus sequence, n = 2). Red melting curves show a sharp positive signal peak when the correct product is amplified and the Eprimer-oBP binds to its target (target 87 genotype A or target 203 genotype F) while non-specific fluorescent signal, below the threshold (blue line) and with a non-sigmoid amplification curve, show a flat melting curve (target 87 genotype F or target 203 genotype A). Black curves are no template negative controls (NC). If amplification curves are hard to interpret, the melting curves can help decide if a signal should be considered positive or negative. [file 12879_2022_7458_MOESM5_ESM.jpg]

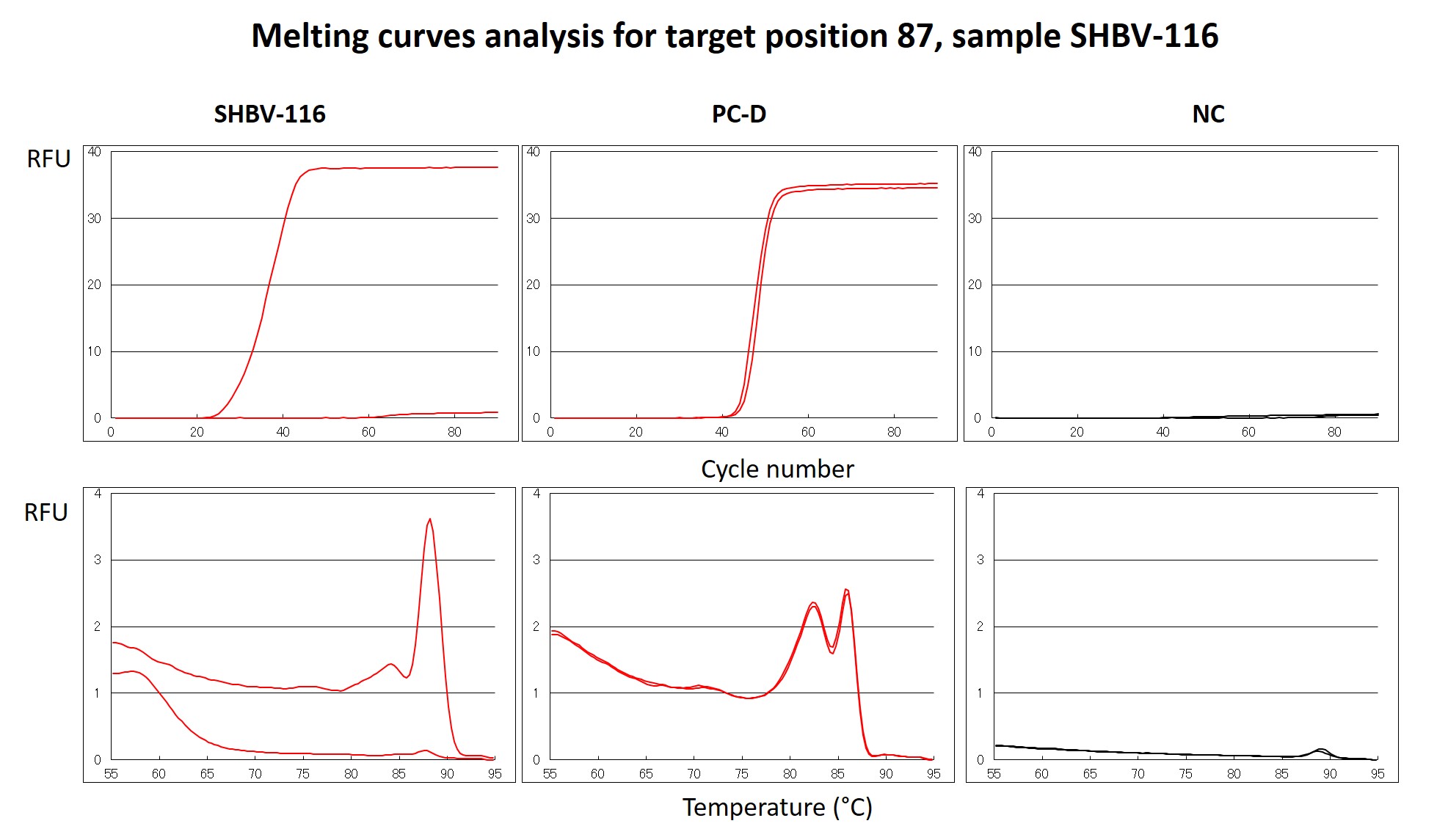

Supplement: Supplementary file 7 — Additional file 7: Melting curves analysis for target position 87, sample SHBV-116. Test on human serum sample SHBV-116, (n = 2). Red melting curves show a sharp positive signal double-peak when the correct product is amplified and the Eprimer-oBP binds to its target as is shown in the positive control genotype D (PC-D) while non-specific fluorescent signal shows a strikingly differently shaped melting curve in SHBV-116. Black curves are no template negative controls (NC). If amplification curves are hard to interpret, the melting curves can help decide if a signal should be considered positive or negative. [file 12879_2022_7458_MOESM7_ESM.jpg]
