# Supplementary material for: SEB genotyping: SmartAmp-Eprimer binary code genotyping for complex, highly variable targets applied to HBV
Source: BMC Infect Dis. 2022 Jun 3;22:516. doi: 10.1186/s12879-022-07458-4 (PMC9164387; doi:10.1186/s12879-022-07458-4)

Data obtained during the development of our primer sets.

- Additional figure 2: Preliminary results on limit of detection.

Preliminary results had shown that the volume of reaction (25µl vs 50µl) as well as the concentration of template purified from cell lines infected with HBV or purified from patient serum samples affected the amplification signal.

In 50µl reaction volume, a serially diluted template of viral particles extracted from cell culture supernatant (either Hep2.2.15.7 or HBP25) yielded reproducible positive amplification curves down to a concentration of 1000 or 750 copies/reaction, respectively (Additional figure 2 A and B). In 25µl reaction volume, Hep2.2.15.7 cell line supernatant at a concentration of 1.0×10^4 copies/reaction yielded 2 out of 3 positive amplification curves and a concentration of $1.0 \times 10^{3.}$ copies/reaction yielded 3 out of 3 positive amplification curves (Additional figure 2C). Three different human serum samples in 50µl reaction volume at a concentration of 0.75×10^5 copies/reaction and 0.75×10^4 copies/reaction each yielded 2 out of 2 positive amplification curves (Additional figure 2D). To avoid diluting the 9 patient serum samples tested in this manuscript, we hypothesized that human serum samples in 25µl reaction volume at a concentration of 1.25×10^5 copies/reaction would yield satisfying results.

The above results to determine the limit of detection have a few limitations, as only one primer set (for target 203), slightly modified with a shorter Eprimer to avoid the On/Off effect, was tested and not all primer sets. All primer sets described in the main part of this manuscript were optimized using plasmid DNA at a concentration of 1.0×10^4 copies/reaction in 25µl reaction volume. A true limit of detection should be tested in a clinical study using over 20 human samples at various concentrations to be significant and offer conclusive results. Determining the limit of detection of the HBV genotyping was not in the scope of this study.

A/ Dilution series of cell culture supernatant: HepG2.2.15.7

Dilution series in DW
HepG2.2.15.7 (cell line infected with genotype D)
NC: DW

1	HepG2-2-15-7 HBV	2.00E+09 copy/mL	1.00E+07 cop/rxn
2	HepG2-2-15-7 HBV	2.00E+08 copy/mL	1.00E+06 cop/rxn
3	HepG2-2-15-7 HBV	2.00E+07 copy/mL	1.00E+05 cop/rxn
4	HepG2-2-15-7 HBV	2.00E+06 copy/mL	1.00E+04 cop/rxn
5	HepG2-2-15-7 HBV	2.00E+05 copy/mL	1.00E+03 cop/rxn
6	NC	0 copy/mL	0 cop/rxn

Differences from the reactions in the manuscript:

- Reaction volume 50µl (vs 25µl in the manuscript)
- Reaction at 65°C (vs 67°C in the manuscript)
- Primer set for target 203 with a shorter Eprimer to avoid the On/Off effect

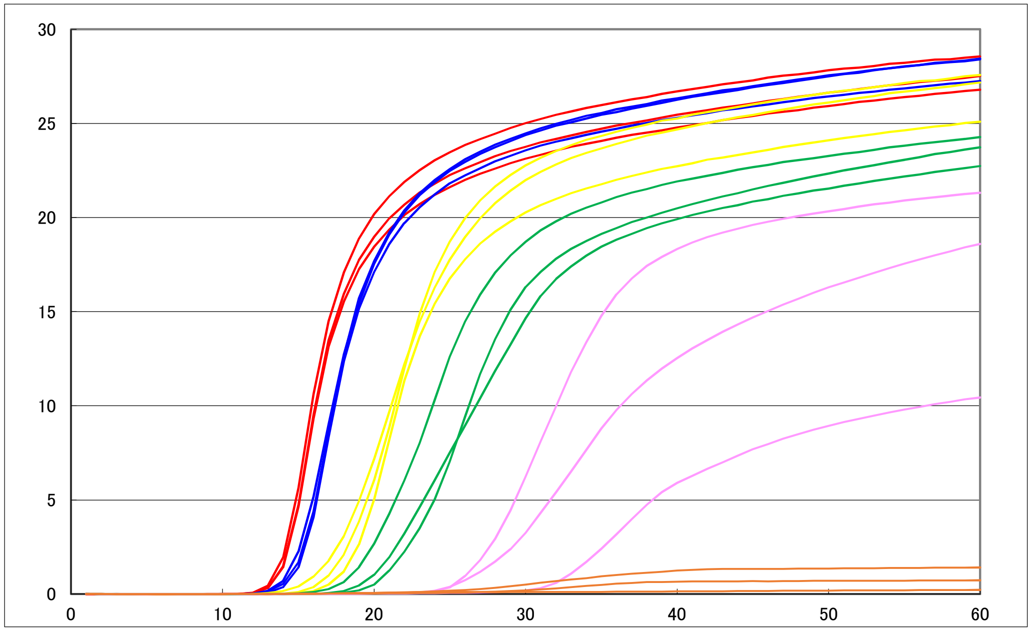

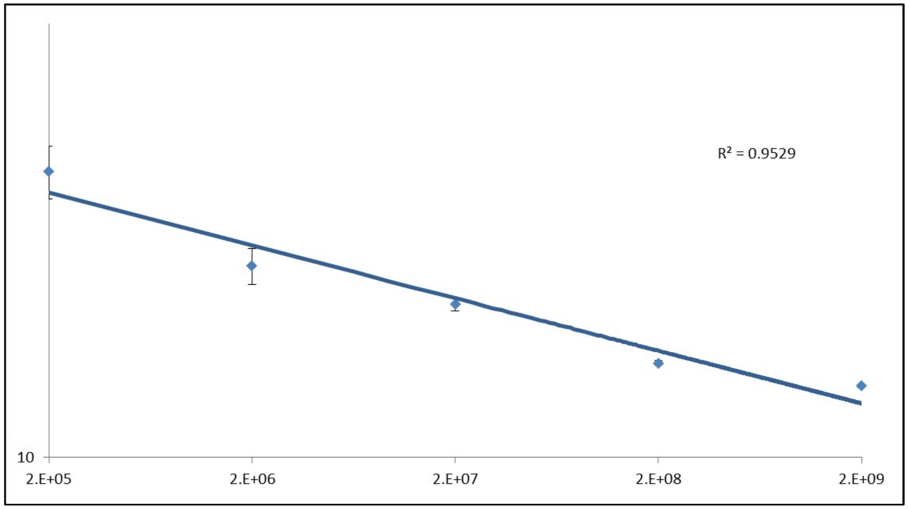

B/ Dilution series of cell culture supernatant: HBP25

Dilution series in DW
HBP25 (cell line infected with genotype C)
NC: DW

Differences from the reactions in the manuscript:

- Reaction volume 50µl (vs 25µl in the manuscript)
- Primer set for target 203 with a shorter Eprimer to avoid the On/Off effect

1	HBP25 HBV	1.00E+07 copy/mL	75000 cop/rxn
2	HBP25 HBV	1.00E+06 copy/mL	7500 cop/rxn
3	HBP25 HBV	1.00E+05 copy/mL	750 cop/rxn
4	HBP25 HBV	1.00E+04 copy/mL	75 cop/rxn
5	HBP25 HBV	1.00E+03 copy/mL	7.5 cop/rxn
6	HBP25 HBV	1.00E+02 copy/mL	0.75 cop/rxn
7	HBP25 HBV	1.00E+01 copy/mL	0.075 cop/rxn
8	NC	0 copy/mL	0 cop/rxn

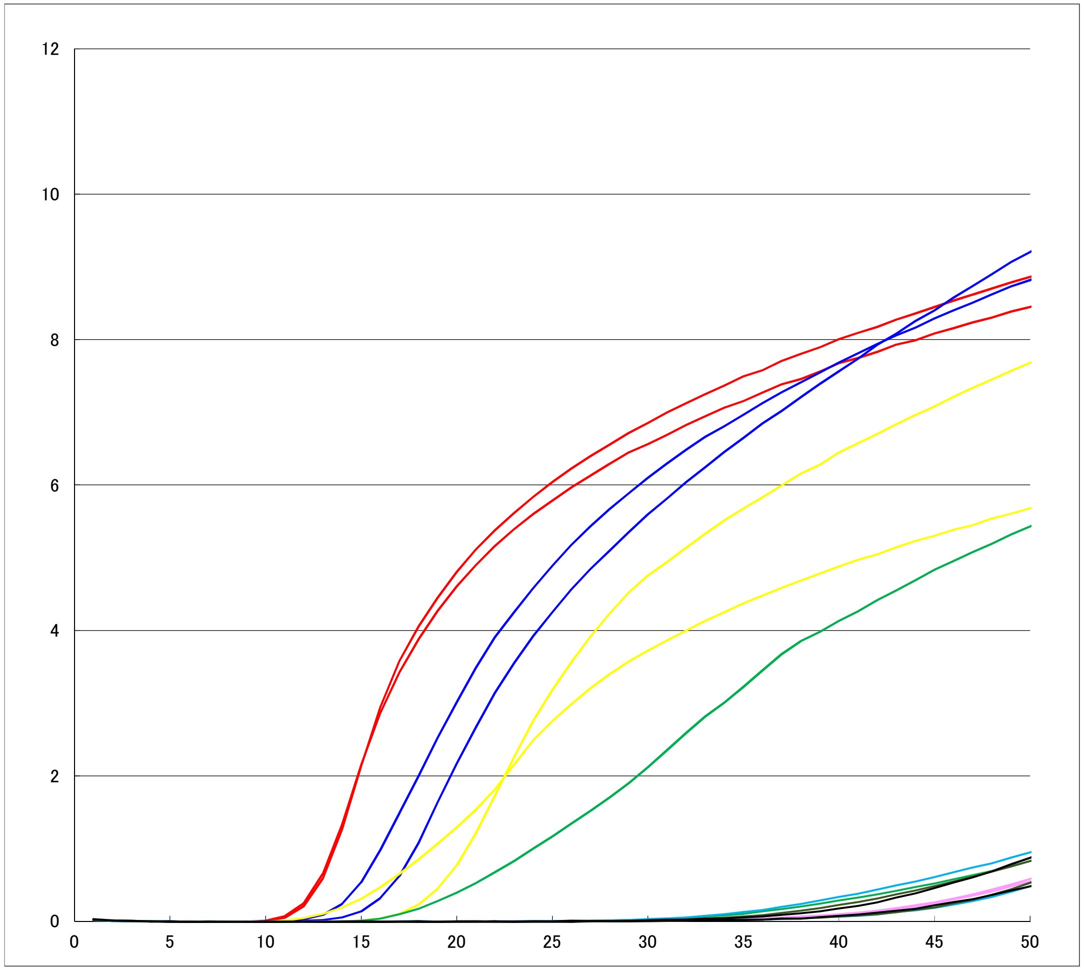

C/ Reaction volume 25µl vs 50µl

1	HepG2-2-15-7 HBV	2.00E+06	copy/mL	1.00E+04	cop/rxn
2	HepG2-2-15-7 HBV	2.00E+05	copy/mL	1.00E+03	cop/rxn
3	HepG2-2-15-7 HBV	2.00E+04	copy/mL	1.00E+02	cop/rxn
4	NC	0	copy/mL	0	cop/rxn

Although loading the same concentrations, the reaction volume impacted the amplification signal.

Dilution series in DW
HepG2.2.15.7 (cell line infected with genotype D)
NC: DW

Differences from the reactions in the manuscript:

- Reaction volume 50µl (vs 25µl in the manuscript)
- Reaction at 65°C (vs 67°C in the manuscript)
- Primer set for target 203 with a shorter Eprimer to avoid the On/Off effect

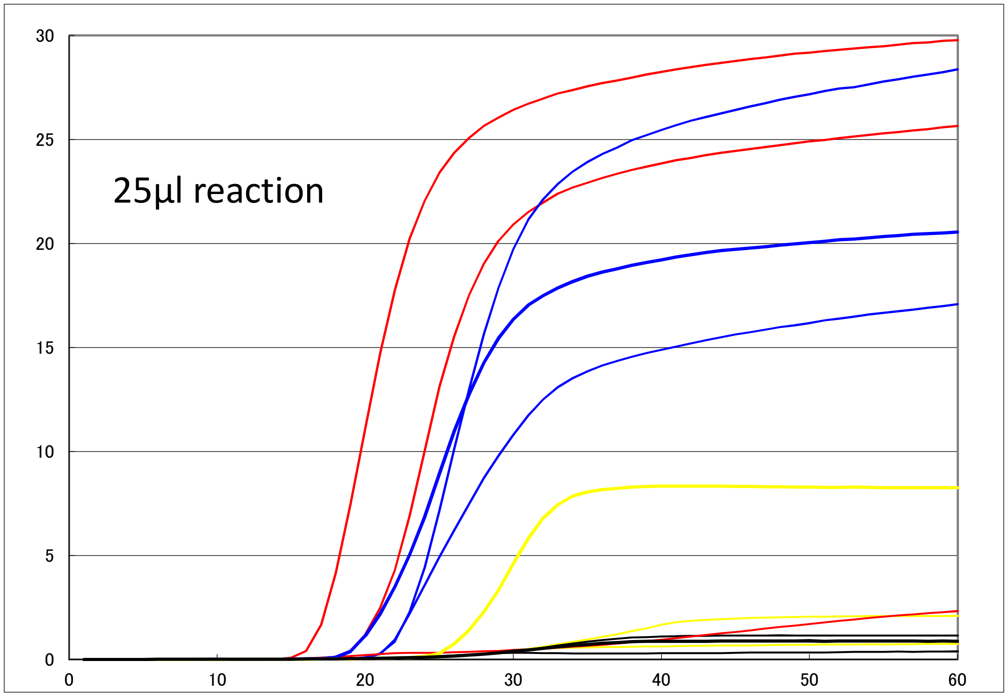

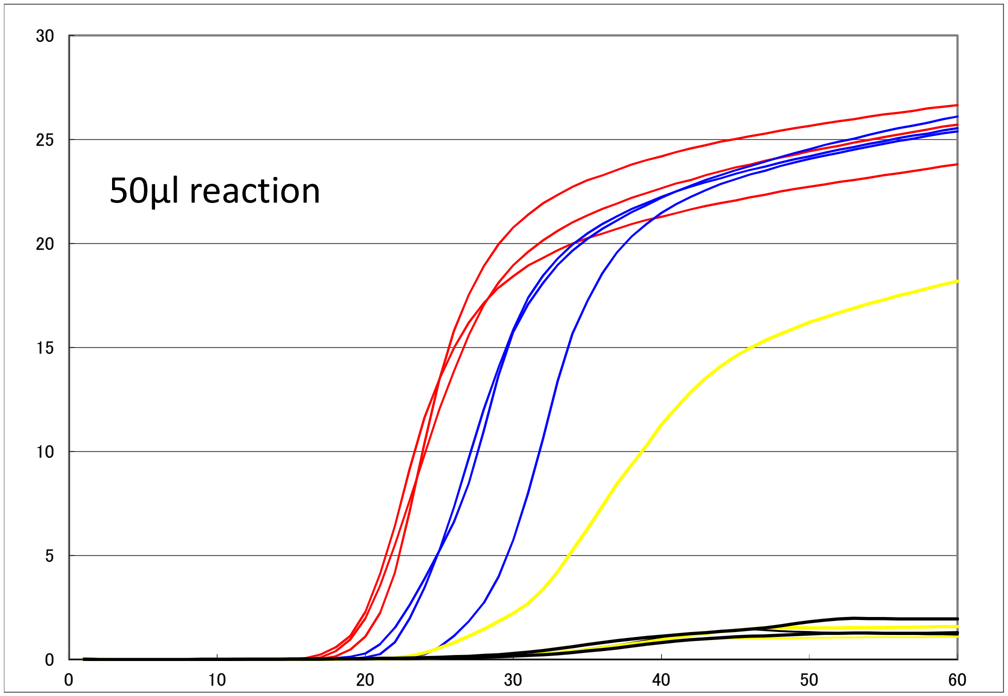

D/ Detection limit in patient serum samples

Infected human serum: 5ul at 10^8cop/ml (all 3 are genotype C)
Dilution series in healthy human serum
PC: HepG2.2.15.7 (cell line infected with genotype D)
NC: Healthy Human serum

1	Human serum HBV	1.00E+07 copy/mL	75000 cop/rxn	
2	Human serum HBV	1.00E+06 copy/mL	7500 cop/rxn	
3	Human serum HBV	1.00E+05 copy/mL	750 cop/rxn	
4	Human serum HBV	1.00E+04 copy/mL	75 cop/rxn	
5	Human serum HBV	1.00E+03 copy/mL	7.5 cop/rxn	
6	NC	0 copy/mL	0 cop/rxn	
7	NC	0 copy/mL	0 cop/rxn	
8	HepG2.2.15.7	1.00E+07 copy/mL	75000 cop/rxn	Dashed line

Differences from the reactions in the manuscript:

- Reaction volume 50µl (vs 25µl in the manuscript)
- Primer set for target 203 with a shorter Eprimer to avoid the On/Off effect

SHBV-016

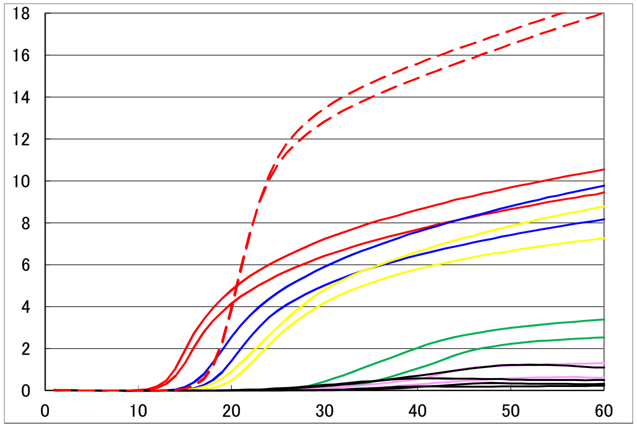

SHBV-140

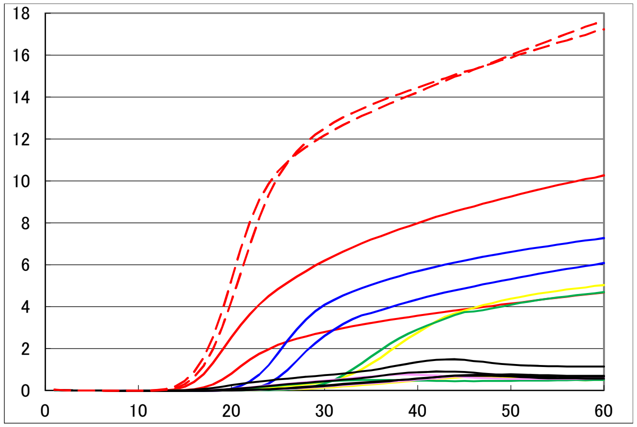

SHBV-165

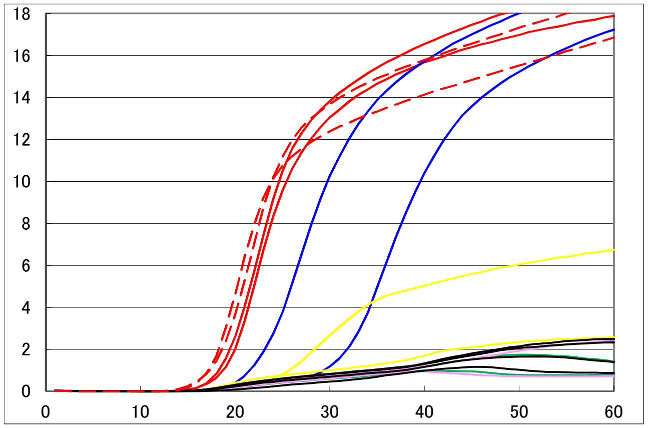

Supplement: Supplementary file 6 — Additional file 6: Preliminary results on limit of detection. We tested various reaction volumes and template concentrations to roughly determine the detection limit of our assays. These results are not exhaustive and do not follow the exact same protocol as used in the main body of our manuscript and should be considered preliminary. [file 12879_2022_7458_MOESM6_ESM.pdf]
